# Changes in Spatiotemporal Dynamics of Default Network Oscillations between 19 and 29 Years of Age

**DOI:** 10.3390/brainsci14070671

**Published:** 2024-06-30

**Authors:** Thorsten Fehr, Sophia Mehrens, Marie-Christine Haag, Anneke Amelung, Kilian Gloy

**Affiliations:** 1 Institute for Psychology, University of Bremen, 28357 Bremen, Germanyk.gloy@uni-bremen.de (K.G.); 2 Center for Advanced Imaging, University of Bremen, 28357 Bremen, Germany

**Keywords:** EEG, resting state, default mode network, neural oscillations, source model, brain dynamics

## Abstract

The exploration of functional resting-state brain developmental parameters and measures can help to improve scientific, psychological, and medical applications. The present work focussed on both traditional approaches, such as topographical power analyses at the signal space level, and advanced approaches, such as the exploration of age-related dynamics of source space data. The results confirmed the expectation that the third life decade would show a kind of stability in oscillatory signal and source-space-related parameters. However, from a source dynamics perspective, different frequency ranges appear to develop quite differently, as reflected in age-related sequential network communication profiles. Among other discoveries, the left anterior cingulate source location could be shown to reduce bi-directional network communication in the lower alpha band, whereas it differentiated its uni- and bidirectional communication dynamics to sub-cortical and posterior brain locations. Higher alpha oscillations enhanced communication dynamics between the thalamus and particularly frontal areas. In conclusion, resting-state data appear to be, at least in part, functionally reorganized in the default mode network, while quantitative measures, such as topographical power and regional source activity, did not correlate with age in the third life decade. In line with other authors, we suggest the further development of a multi-perspective approach in biosignal analyses.

## 1. Introduction

### 1.1. About the Functional Neuroanatomy of the Human Default Mode Network (DMN) and Its Developmental Changes

One of the problems with which the neuroscientist is confronted while examining brain physiological changes over time is their non-linear developmental nature [1,2,3,4,5,6]. Furthermore, individual factors, as well as contextual and learning histories, were discovered largely to modulate the elaboration of complex perception–action networks, particularly in the heteromodal association cortices [7]. Anatomical properties such as symmetrically closed (anisotropic) and asymmetrically open (isotropic) field cytoarchitectonic [8] and related physiological parameters, such as, for example, local field potentials and brain oscillations in different frequency ranges and neural sub-systems [5,9], were discovered to substantially modulate brain function and oscillations via dynamic neural network interaction [10]. There is evidence that oscillatory brain activity is modulated by white [11] and grey [12] matter plasticity/maturation, and/or vice versa, which would also, at least in part, explain the substantial changes in (resting-state) brain oscillations during healthy and pathological development.

Electroencephalography (EEG) and magnetoencephalography (MEG) provide powerful scientific and non-invasive tools for the examination of fast, dynamic brain oscillatory fluctuations and their changes during brain development. Popov et al. [13] reported good and very good retest reliability for several resting-state EEG parameters, such as power estimates on both scalp and source levels, individual alpha peak power and frequency, microstate measures, and connectivity parameters. Whereas at the signal-space level (i.e., topographical signals at electrode positions from standardized scalp locations), data mainly provide information of high temporal resolution, source space analyses provide less spatially smeared but also highly temporal information [14]. However, EEG source space analyses confront us with the so-called inverse problem [15]. As discrete source analyses are based on approximation algorithms, there is an infinite number of solutions that can explain a particular scalp topography. In the present work, we suggest, therefore, a hypothesis-driven ad hoc model [14,16,17,18] to be seeded into source space for further spatiotemporal analyses of band-pass filtered data in different pre-defined frequency ranges.

There is a variety of seminal work on changes in oscillatory brain activity over the life span, and these changes appear to be of a rather non-linear/ordinal nature [13,19,20,21], accompanied by more or less dramatic reorganization and maturation of the neural network architecture, such as prominent phases of dendritic proliferation (e.g., during childhood), pruning (e.g., during adolescence), and myelinization (i.e., till the end of the third life decade), progressing predominantly from primary to heteromodal association cortices [3,4,6,22]. Most prominently, resting-state alpha oscillations showed a peak around ten Hz at posterior electrode sites in most individuals (e.g., [23,24,25]). Whereas alpha peak activation has been reported to be intra-individually stable on the short-term scale [26], it appears to increase from childhood to adolescence [19,27,28], shows a plateau during early adulthood, and decreases again in later adulthood. Multi-channel MEG data were reported to reveal larger general absolute power values in adolescents as compared to adults in delta and theta, as well as lower alpha power (1.5–10.5 Hz), lower beta frequency band power (13.0–21.5 Hz), and larger higher alpha power (10.5–13.0 Hz) at frontal and frontotemporal sensor sites [20]. Adolescents showed relatively larger relative power values in delta and theta bands and lower relative power values in alpha, beta, and gamma frequency ranges. Cross-sectional correlations within groups showed a regional decrease in absolute delta and theta in both adolescents and adults and a general decrease in absolute lower alpha power in adolescents. Absolute beta and lower gamma band power partially decreased mainly in adults, and less prominently in adolescents. Higher absolute gamma power (65–100 Hz) increased over central sites prominently in adults. Relative power values showed a general decrease in delta and an increase in beta frequency ranges in adults and a decrease in relative lower alpha band power in adolescents. Summarizing, it appears that brain oscillations across childhood, adolescence, and early and late adulthood change in a non-linear—or to say non-uniform—manner, whereas the third life decade (i.e., during the twentieth) was discovered to show a kind of plateau in oscillatory brain development [13]. In the present work, we addressed this question from different exploratory perspectives to stimulate further prospective hypotheses building and testing.

Brain areas discovered to be involved in the DMN were discovered to become especially active during the mental resting state, which was associated with different kinds of internal mental states, such as introspection and mind wandering [29,30], but also self-referential thought [31,32]. It also appears that DMN activity decreases during tasks with external stimulation and demands (e.g., [33]), which would make the DMN a complementary counterpart for perceptually and executively driven mental processes. Functional neuroimaging approaches revealed differences in neural network dynamics (e.g., in connectivity) across age during adulthood [34,35,36], especially involving communication between anterior and posterior parts of the DMN, with medial prefrontal areas serving as an important neural node [37,38,39].

Chow et al. [40] speculated that because most work on age-related neural network dynamics in the DMN came from functional neuroimaging studies (i.e., fMRI and PET), the discussion might predominantly be directed towards slower network activation (i.e., as functional neuroimaging approaches mostly relied on slow haemodynamic brain responses), while higher frequency dynamics might not be considered as much as they should [41,42]. Approaches with higher temporal resolution, such as EEG and MEG, should come more into play for the examination of age-related changes in higher-frequency-related network dynamics (e.g., [40]). However, as Chow and colleagues [40] critically mentioned, most EEG work on age-related resting-state network dynamics was based on signal space data (i.e., at electrode sensor level and, respectively, smeared surface topographies), leading to valuable but quite heterogeneous findings (e.g., [43,44,45,46]) on the basis of different methodological approaches for the determination of respective neural network dynamics in theta, alpha, and beta frequency ranges (e.g., [43,44,45,46,47,48]). Chow et al. [40] used source space data to explore age-related differences in EEG phase synchronization of the DMN in resting-state (between one and 50 Hz), particularly between medial prefrontal areas and other brain regions of the DMN. The reported data reflected a decrease in higher alpha and an increase in theta and beta frequency band connectivity between medial prefrontal and other brain areas in the DMN, indicating, according to the author’s conclusions, a non-uniform frequency depending on the nature of the neural DMN dynamics [40].

### 1.2. Which Brain Areas Shall Be Included in an Ad Hoc Model for the Exploration of Respective Neural Generator Dynamics in the Default Mode Network (DMN)?

#### 1.2.1. Neurophysiological Mapping of the Default Mode Network DMN

One has to seriously differentiate between structural and functional brain networks. Brain development changes, especially during the first three life decades, while structural sprouting, pruning, and myelinization follow non-linear trajectories with potential modulatory influence on functional development [3,4,6,7,22,49]. Together with individually experienced context factors and respective learning histories, this appears to massively shape mental perceptual and conceptual—or differential psychological—processing concepts at different complexity levels. This has been discussed in relation to an elaboration of neural perception–action cycle networks representing stereotypes of individual cognitive style and mental strategy [7,22,50,51]. Both (micro-)structural and (psycho-)functional development might be discussed in relation to a kind of default network readiness of the brain across individual development. Involved neural structures in the DMN have been identified by examining their functional involvement, particularly during mental resting condition. Association cortices and networks with particular structural connectivity have been discussed as important neural instances [31,52]. It has been shown that regions within the DMN are part of a group of nodes that have stronger connections with each other than with other regions of the cortex [53]. For example, the precuneus was identified as one such potentially important hub [52], which is in line with the discussion of the precuneus as part of the so-labeled heteromodal association cortices (HACs), which become more and more involved during individual learning history for the neural elaboration of complex cognitive and emotional mental processing [7,22,51,54]. The precuneus is strongly and reciprocally connected to different association areas, such as the superior parietal lobule, temporal polymodal cortices, and prefrontal lobe structures. These brain areas were discovered to be important for both the functional organization of the brain and the DMN, as they coordinate multiple streams of information also related to other heteromodal and paralimbic association cortices that have been discovered to provide relevant neural instances for the integration of multiple streams of cognitive and emotional information [52].

Additional sub-network units have been discovered to be involved in the DMN, such as, for example, the frontoparietal network (FPN) and the dorsal attention network (DAN) [55]. FPN, DAN, and HAC networks were discovered to be interactively involved in state-dependent changes in younger and older adults. Functional connectivity between these networks at rest and during cognitive demands (i.e., during the multi-source interference task, MSIT) showed differences in the interactions in the FPN and DAN and between these sub-networks and the DMN. In particular, the FPN was more strongly coupled with the DMN at resting, while the DAN was stronger recruited during MSIT processing in both age groups [56]. The increased interaction between the FPN and the DMN during resting-states suggests that frontal brain regions modulate the activity of the DMN, especially when the brain switches to an internal processing mode. In the following sub-sections, a network of particular brain structures is discussed as a series of potentially important neural instances for the regulation of the DMN.

#### 1.2.2. Frontal Brain Structures and the Default Mode Network (DMN)

Brain structures discovered to be involved in the DMN have also been discovered to be recruited during autobiographical, social, and emotional task processing [57]. Connectivity between right inferior frontal gyrus and several DMN areas in the left hemisphere were related to emotional interference processing, regulating, among other things, the interplay between internal and externally driven network activities [58]. It has also been discovered that the medial superior frontal gyrus and the middle frontal gyrus potentially provide important nodes in the DMN [57,59], and the ventromedial prefrontal cortex (vmPFC) has been discovered to be involved in ongoing and non-externally driven thought patterns, particularly those with episodic or social content [57,60]. The vmPFC appears to be also involved in the suppression of stimulus-related tasks associated with the DMN [59,61]. The anterior ventrolateral inferior frontal cortex [61] and the anterior cingulate cortex (ACC) has also been discussed as parts of the DMN [59,62,63,64,65,66]. Koch and colleagues [66] used fMRI data to investigate the strength of the interconnectivity of DMN regions and the extent of DMN coactivation. A conjunction analysis across twelve fMRI studies further illustrates the role of the ACC in task-related deactivation [66].

#### 1.2.3. Parietal and Occipital Brain Structures and the Default Mode Network (DMN)

The posterior cingulate cortex (PCC) has repeatedly been shown to be connected to other areas of the DMN [61,65,67,68,69]. It appears as one of the nodes that shows the highest functional coactivation with other regions of the DMN, such as the ACC, the superior frontal gyrus, and the hippocampus [70]. A significant reduction in this coactivation in the PCC was observed in late adulthood [65], which could analogously be found for the right precuneus [59,67,70,71]. Davey et al. [72] discussed the inferior parietal lobule (IPL) as one of the key structures of the DMN that was related to activation patterns in the ventral posterior cingulate (PCC) and the medial prefrontal cortex (medPFC) during rest compared to situations requiring external attention. The IPL could also be related to processes of self-referencing [71]. The intraparietal sulcus (IPS) was discussed as a major instance in spatial orientation and visual attention [73], but it was also discussed as an important part of the DMN [57]. The caudal IPS has further been reported to show functional connectivity to several cortical areas, including the lateral and medial prefrontal cortex, the posterior cingulate cortex, the anterior middle frontal cortex, the inferior temporal cortex, and the parahippocampal gyrus [74,75].

Wu et al. [76] observed that better task performance was associated with increased suppression of irrelevant information in the PCC, in occipitotemporal cortex areas, and the extrastriate cortex, which has been concluded to be consistent with the generally known assumption about the function of the DMN [76].

#### 1.2.4. Temporal Brain Structures and the Default Mode Network (DMN)

Both lateral and medial temporal brain areas were related to the DMN. A reduction of cognitive load during decision making has been related to an increase in activation in medial temporal areas but also in particular parietal and frontal brain regions [77]. Based on resting-state connectivity analyses, Briggs and colleagues [78] discussed the middle temporal gyrus from posterior parts to the anterior pole as an integrative instance of the DMN modulating semantic processing (i.e., learning and, in particular, verbal memory) and language production. Furthermore, medial middle temporal gyrus was discovered to be involved in the modulation of semantic memory and control networks [79]. These and other findings emphasize the assumption that the MTG plays a central role in integrating automatic retrieval in the default mode network with cognitively demanding, goal-directed thinking tasks and serves as a central hub in the DMN [57,70].

#### 1.2.5. Subcortical Brain Structures and the Default Mode Network (DMN)

The regions that are part of the default mode network (DMN) are reported inconsistently across studies. One of the regions that is not always associated with the DMN is the thalamus, with its manyfold specific and unspecific nuclei and connections to numerous cortical and sub-cortical brain networks. The thalamus acts as a central hub in the brain that receives sensory information from various sensory organs and relays it to the appropriate regions of the brain, including areas associated with the DMN. Furthermore, the thalamus also sends feedback signals to sensory regions, emphasizing its role in modulating cortical activity and integrating information [80]. Additionally, the thalamus has been shown to connect with other key regions of the DMN, such as the posterior cingulate cortex. These anatomical and functional connections between the thalamus and core areas of the DMN suggest that the thalamus plays an essential role in coordinating the activity of the DMN and should therefore be considered in a respective DMN model [61,80,81,82,83]. Furthermore, thalamus nuclei were associated with episodic memory processing and spatial navigation, which were also associated with activations of the DMN [57,84]. The hippocampus is an additional structure that should be considered in a DMN model as functional connectivity studies provided evidence for its functional coupling to DMN-structures such as the precuneus and both the dorsal and the ventral mPFC [81].

#### 1.2.6. Can Particular Brain Areas Be Excluded from the Default Mode Network (DMN)?

From an equipotentiality and holistically driven standpoint, it appears inappropriate to exclude particular brain areas from any kind of more or less complex mental process, but from a modularity point of view, it would be appropriate [22,85]. Thus, Fehr [22] suggested a hybrid strategy when discussing local neural network involvement during the processing of any kind of information processing, but particularly the organization of complex mental processing, which was discovered to be reflected in terms of HOW information was processed, not WHAT concrete information [5,9,22,50,51]).

Smallwood et al. [77] suggested a model for the DMN that predominantly includes those brain areas which show the highest distance to the so-called topographical, primary cortices, such as primary and retinotopically organized visual, tonotopically organized auditory, and body landmark (i.e., homunculus)-oriented somatotopic, and primary motor cortices. Later, primary cortices were also discovered to host so-called phyletic memories, from which all other memories emerge to secondary and heteromodal cortices during individual learning histories [50,51]. Analogously, Fehr [22] further suggested developmental starting nodes in the middle and superior, medial frontal, and intra-parietal areas, which he discussed to provide important neural instances, from which complex mental processing performance (i.e., executive, working memories, integrative information processing, canonical, spatial coordination, etc.) emerges and plastically recruits more and more heteromodal, adjacent brain areas during individual development. Patterns of activation during complex mental processing were discovered to reflect individual mental strategies (i.e., the HOW) applied during task processing. Furthermore, many studies using too-restrictive statistical alpha-error correction might predominantly report a kind of by-product activation pattern, relying on these developmental starting points and not the relevant adjacent and more individually recruited task-related neural resources [86]. Latter developmental neural starting points might also provide good candidates to be considered for a DMN model. In Section 2.3.2, respective anatomical center coordinates are defined and listed in detail.

### 1.3. The Present Work, Exploratory Questions, and Several Working Hypotheses

Data reported so far imply that there is a lot more to do in terms of the exploratory qualification of the nature of age-related neural network dynamics in the DMN. The present work addresses changes in brain oscillations and the follow-up dynamics of respective regional source equivalents during the third life decade via cross-sectional correlation analyses. Here, we followed both a traditional and a data-driven exploratory approach. Both topographical signal and ad hoc modeled source space network data were related to age and several state- and trait-related covariates such as ratings of current psychological and physiological well-being, acute stress level, and several personality scales. The following main hypotheses and exploratory questions were addressed:

(1) It was expected that both relative and absolute power spectra peak at around 10 Hz with topographical dominance over posterior brain areas.

(2) Both absolute and relative topographically distributed power spectra were expected to substantially corelate with age over the third life decade.

(3) The carefully deduced default mode network source model (DNMSM, see Section 2.3.2 for technical details) will produce less than 10% residual variance when applied to band-pass-filtered single epoch signal space data in different traditionally defined frequency ranges between one and 35 Hz (i.e., delta, theta, alpha low and high, beta low and high, and gamma).

(4) Follow-up communication dynamics in the DNMSM were explored, and it was expected that different frequency band oscillations might produce different profiles of functional changes across the third life decade. The present data shall provide a hypothetical basis for subsequent studies considering different mental functions, age-cohorts, and pathological conditions.

## 2. Materials and Methods

For the present work, both traditional and data-driven exploratory approaches were considered. In a first step, resting-state EEG data from two laboratories were analyzed via fast Fourier transform and topographical correlation analyses with age and several state and trait variables. In a second step, a default-mode network-oriented source model was introduced and applied to explore potential age-related changes in neurophysiological dynamics in resting-state EEG data across the third life decade.

### 2.1. Sample characteristics

Resting-state EEG data and several test-scale values of 54 participants from two different studies on similar social decision making tasks (not published so far) were obtained and analyzed. The used experimental setups were designed according to the Code of Ethics of the World Medical Association (Declaration of Helsinki, published in the British Medical Journal, 18 July 1964), and stimulus materials were approved by the local ethics committee. The participant group consists of 39 male and 15 female individuals (range: 19 to 29; mean age: 23.7+/−2.8 years, with four 19-, four 20-, four 21-, nine 22-, five 23-, ten 24-, two 25-, three 26-, six 27-, six 28-, and one 29-year-old participants). All of them were right-handed, as determined by the Edinburgh Inventory [87], and all participants gave written informed consent with respect to their participation and did not report any chronic psychological or physiological disorders. Before the examination, participants were asked for psychological, neurological, and other severe and/or chronic diseases to exclude individuals with the respective pathological backgrounds from the sample. Thus, in the present work, only healthy young adults (all of them university students) in their third life decade (i.e., between 19 and 29 years, see also above) of brain development were included as participants.

### 2.2. Experimental Procedures

Different variable categories were considered in the present work: AGE (in years) was included as the most important independent variable. The state variables PSYCH (acute psychological well-being), PHYS (acute physiological well-being), and STRESS (acute stress level) were assessed by ten-leveled self-rating Likert scales (1–10). The so-called “big five” personality trait dimensions—Neuroticism, Extraversion, Openness to Experience, Agreeableness, and Conscientiousness—were assessed with the 60-item NEO-FFI-inventory, introduced by Costa and McRae [88] (German version by [89]).

Resting EEG data included in the present work were measured after participants performed a series of quasi-naturalistic social decision tasks [90]. During rest, participants were asked to watch a smoothed dot in the center of a black screen in front and wipe all thoughts that come into their mind beside. All participants reported that they could easily do that and that this relaxing phase was a welcome break after the previous experimental runs.

In both laboratories, participants were familiarized with the measurement environment, and recordings were performed in a dimly lit and comfortably heated room without any noise contamination.

### 2.3. Multi-Channel-Electroencephalography

Data from two different laboratories were included, both using a comparable 64-electrode measurement setup. In the first laboratory, at Bremen University, EEG recording was conducted with 64 Ag/AgCl scalp electrodes placed according to the extended international standardized 10-10 system (data were measured average referenced). These electrodes included FP1, FP2, AFz, AF3, AF4, AF7, AF8, Fz, F1, F2, F3, F4, F5, F6, F7, F8, FCz, FC1, FC2, FC3, FC4, FC5, FC6, FC7, FC8, Cz, C1, C2, C3, C4, C5, C6, T7, T8, CPz, CP1, CP2, CP3, CP4, CP5, CP6, TP7, TP8, TP9, TP10, Pz, P1, P2, P3, P4, P5, P6, P7, P8, POz, PO3, PO4, PO7, PO8, PO9, PO10, Oz, O1, and O2 positions. In both participating laboratories, an electro-oculogram (EOG) was recorded with four additional Ag/AgCl electrodes that were attached to the left and right canthi (horizontal EOG) and below and above the right eye (vertical EOG). The ground electrode was placed on the left masseter muscle near the chin. The EEG data were recorded with EEmagine software (version 3.3). The signal was amplified (REFA 136 multi-channel system; TMS international) and digitized with a sampling rate of 512 Hz. The impedance of each channel was kept below 10 kOhm.

In the second laboratory, at the University of Cologne (Germany), EEG data were recorded with 64 Ag/AgCl scalp electrodes distributed over the scalp according to the 10-10 system (Cz, FC2, CP2, CP1, FC1, F4, C4, P4, Pz, P3, C3, F3, Fz, FC6, CP6, CP5, FC5, FP2, F8, T8, P8, O2, Oz, O1, P7, T7, F7, FP1, TP10, PO10, PO9, TP9, FCz, C2, CPz, C1, F2, FC4, CP4, P2, P1, CP3, FC3, F1, AFz, AF4, F6, C6, P6, PO4, POz, PO3, P5, C5, F5, AF3, AF8, FT8, TP8, PO8, PO7, TP7, FT7, AF7) and using a machine by BioSemi; the model was ActiveTwo AD-box ADC-12. Data were digitized with a sampling rate of 512 Hz. The default reference electrodes were CSM and DLR. The recorded data are always referenced to these electrodes; during the processing of the data, however, the reference was changed to average reference. EEG data were recorded with the software ActiView 720 LoRes. The ground electrode was placed on the left cheek near the chin.

#### 2.3.1. Signal Space: Topographical Fast Fourier Analyses (Absolute and Relative Power Spectra)

FFT was calculated for continuous data recorded during resting condition, and values were obtained by averaging power spectra revealed by the application of FFT procedures on moving data time segments of 2000 ms (28.8+/−2.7 trials). Each data segment overlapped 50% with the next segment and was multiplied by a cosine squared (cos2) window. This combination of overlap and windowing ensures that each time point contributes equally to the mean spectrum (since cos2(x) + sin2(x) = 1) (see also BESA^®^ -Software, version 5.2.2).

FFT data were divided into seven frequency bands: delta [1.0–4.0 Hz], theta [4.0–8.0 Hz], alpha-low [8.0–10.5 Hz], alpha-high [10.5–13.0 Hz], beta-low [13.0–21.5 Hz], beta-high [21.5–30.0 Hz], and one gamma band [30.0–35.0 Hz]. In addition to the absolute power band analyses, relative spectra were calculated (band-related power values divided by absolute power in the whole spectrum).

After careful visual inspection of the averaged referenced raw data, several channels were interpolated (no more than five electrode positions per data set), and artefact contaminated trials were excluded from further analyses on the basis of individual visual trial-by-trial inspection. About 30 prototypic blinks were averaged for each individual to produce topographical templates for their respective artefact correction implemented in BESA^®^-software (version 5.2.2). The respective procedure follows an adaptive correction strategy. Brain activity is estimated by a defined data epoch (here, an averaged blink epoch as mentioned above). Data epochs were considered to represent brain activity when the correlation between data and artifact topography did not exceed a certain threshold and when the signal amplitudes were below a particular threshold. The remaining data segments served as a basis for a principal component analysis (PCA); identifying all PCA components that explained more than a specified variance level provided the topographical basis for the artefact correction procedure. Through this procedure, the data were decomposed using all respective topographies into a linear combination of brain and artifact activities. Finally, the estimated artifact signals are much less overlapped with brain activity and can be subtracted from the original signals without substantial functional signal loss [91].

For topographical, correlative signal space analyses, FFT data were, respectively, averaged over the following electrode clusters: midline frontal (f: AF3, AFz, AF4, F1, Fz, F2), central (c: FC1, FCz, FC2, C1, Cz, C2), parietal (p: CP1, CPz, CP2, P1, Pz, P2), occipital (o: PO3, POz, PO4, O1, Oz, O2), lateral frontotemporal left (ftl: F7, F5, F3, FT7, FC5, FC3), frontotemporal right (ftr: F4, F6, F8, FC4, FC6, FT8), parietotemporal left (ptl: T7, C5, C3, TP7, CP5, CP3), parietotemporal right (ptr: C4, C6, T8, CP4, CP6, TP8), occipitotemporal left (otl: P7 P5 P3 P9 PO7), and occipitotemporal right (otr: P4 P6 P8 PO8 P10).

#### 2.3.2. Source Space: Default Network Model for Ad Hoc Seeding Procedures

**Figure 1:**
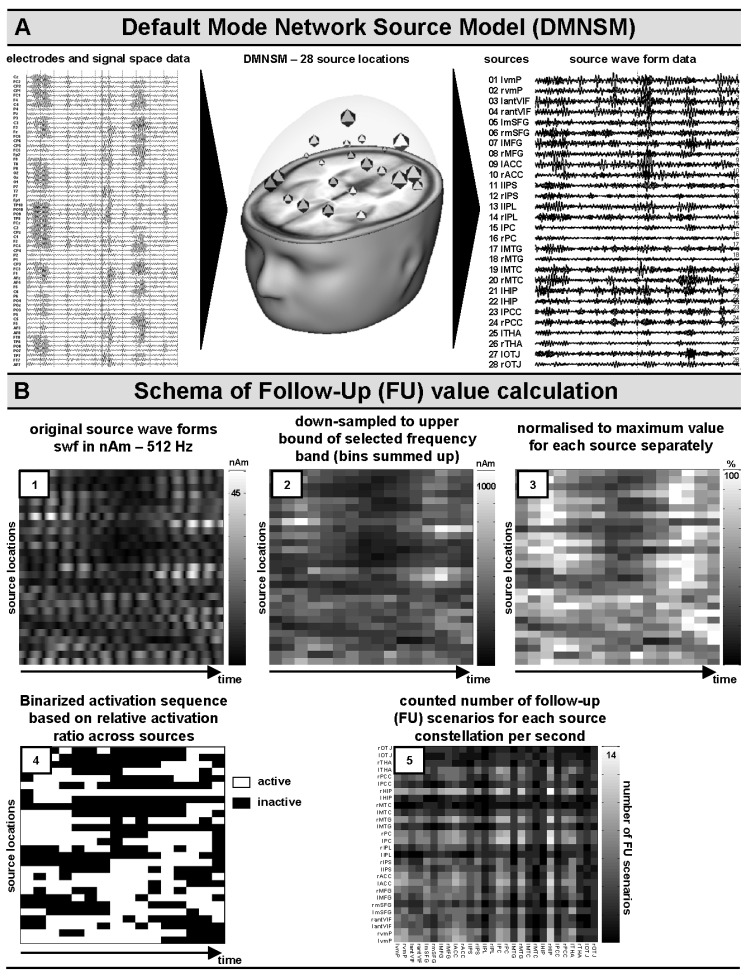
Panel (**A**): illustration of the appliance of the default network mode source model (DNMSM); panel (**B**): illustration of analysis steps for the determination of source follow-up (FU) frequencies. Please see text for details to processing steps in panels 1–5.

For the present work, we introduced a Default Mode Network Source Model (DMNSM, see Figure 1A, middle panel, and Table 1 for anatomical labels and shortcuts), and respective Talairach center coordinates [92] to be seeded ad hoc [15] and used to obtain source wave forms (in nAm units) from band-pass-filtered data (see Section 2.3.1 for respective bands and frequency borders). This procedure was chosen to circumvent the inverse problem during free source fitting procedures usually based on maximum likelihood algorithms [14,15,18,93,94,95].

The here-proposed Default Mode Network Source Model (DMNSM) was based on prior knowledge derived from functional neuroimaging studies and further elaborate knowledge about discussed issues pertaining to the functional neuroanatomy of the so-labeled Default Mode Network (DMN; [96]). In the following sections, the importance of interdependent neural networks in the DMN addressing particular principal brain structures is elaborated and considered in relation to the above-mentioned DMNSM.

Based on the previous sections and the introduction, the here-introduced Default Mode Network Source Model (DMNSM) comprises 28 approximate center coordinates (according to the Talairach taxonomy [92]) of 14 anatomical structures, in parallel, for the left and right hemispheres (see Table 1 for details and Figure 1A for illustration). This ad hoc model serves as a basis for seeding procedures into band-pass-filtered signal space data. The resulting source wave forms (nAm) were further analyzed for dynamic properties and correlative relationships with AGE and several state and trait variables (see Section 2.3.3 and Section 2.3.4 for more details).

**Table 1:** Number of regional sources (RSs), anatomical-label, short-label, and approximated Talairach center coordinates for their respective RSs to be seeded for further analyses (i.e., Default Mode Network Source Model/DMNSM model).

RS	Anatomical Label	Short Label	TAL-x	TAL-y	TAL-z
1	Ventromedial Prefrontal left	lvmP	−8	30	−24
2	Ventromedial Prefrontal right	rvmP	8	30	−24
3	Anterior Ventrolateral Inferior Frontal left	lantVIF	−28	25	−24
4	Anterior Ventrolateral Inferior Frontal right	rantVIF	28	25	−24
5	Medial Superior Frontal Gyrus left	lmSFG	−10	19	52
6	Medial Superior Frontal Gyrus right	rmSFG	10	19	52
7	Middle Frontal Gyrus left	lMFG	−38	40	20
8	Middle Frontal Gyrus right	rMFG	38	40	20
9	Anterior Cingulate Cortex left	lACC	−10	45	17
10	Anterior Cingulate Cortex right	rACC	10	45	17
11	Intraparietal Sulcus left	lIPS	−35	−60	50
12	Intraparietal Sulcus right	rIPS	35	−60	50
13	Inferior Parietal Lobule left	lIPL	−56	−36	28
14	Inferior Parietal Lobule right	rIPL	56	−36	28
15	Precuneus left	lPC	−15	−58	36
16	Precuneus right	rPC	15	−58	36
17	Middle Temporal Gyrus left	lMTG	−44	−66	17
18	Middle Temporal Gyrus right	rMTG	44	−66	17
19	Medial Temporal Cortex left	lMTC	−55	14	10
20	Medial Temporal Cortex right	rMTC	55	14	10
21	Hippocampus left	lHIP	−31	−25	−8
22	Hippocampus right	rHIP	31	−25	−8
23	Posterior Cingulate Cortex left	lPCC	−10	−58	11
24	Posterior Cingulate right	rPCC	10	−58	11
25	Thalamus left	lTHA	−13	−17	8
26	Thalamus right	rTHA	13	−17	8
27	Occipitotemporal junction left	lOTJ	−44	−71	2
28	Occipitotemporal junction right	rOTJ	44	−71	2

#### 2.3.3. Source Space: Quantification of Dynamic Follow-Up (FU) Generator Activities

For the FU analyses, a 30 s resting-state data period was extracted and divided in 1 s trial epochs. In additional to the eye–artefact correction procedures described in Section 2.3.1, all 1 s trial epochs that contained time bins with more than two standard deviations above the mean nAm values were rejected from further analyses. In the end, there were 26.3 (+/−5.9) 1 s epochs left.

The source model (DMNSM), introduced in Section 2.3.2, was, respectively, applied to band-pass-filtered signal space data (Figure 1A). Afterwards, the resulting source wave forms (SWFs, in nAm units) for each regional source (RS) of the DMNSM were written for further analyses. For the present approach, SWF data (Figure 1B, panel 1) were down-sampled to the double of the upper border (minimum criterion according to the Nyquist theorem) of the respective, pre-defined frequency band (see Section 2.3.1). For each new down-sampled time bin (delta: 8.0 Hz; theta: 16 Hz; alpha low: 22.0 Hz; alpha high: 26.0 Hz; beta low: 43 Hz; beta high: 60 Hz; gamma: 70 Hz), nAm values were summed up accordingly (Figure 1B, panel 2).

As both the amount of neural generator activities and their contribution to surface topographical mapping depend on depth, local cytoarchitecture (electrical open/closed field constellation) [8], and concepts like sparse coding in neural network communication [97], in the present approach, data were source-wise normalized to the maximum nAm value of each included one-second epoch. Thus, the resulting data further represented percentage values representing dynamic fluctuations without providing absolute amplitude information for a direct comparison between RSs (Figure 1B, panel 3).

In the next step, activation dynamics between sources were determined by estimating the ratio of percentage values of each RS for each time bin separately [98,99]. In cases where the percentage value of a RS represented more than the nth part (*n* = number of RS = 28 in the present DMNSM) of the sum of all RSs, it was labeled as 1 = active (otherwise, as 0 = not active). This procedure resulted in a binary matrix of dynamic activations and deactivations (see Figure 1B, panel 4, for illustration).

Finally, the resulting binary matrices were analyzed with a strictly non-linear and descriptive approach, which was further labeled as the follow-up (FU) analyses. Each active RS (indicated by 1 in the binary matrix) was examined for other active sources on the consecutive following time bin. If so, the respective follow-up constellation between two sources, A and B, was incremented by one, resulting in follow-up matrices (i.e., FU frequencies for 28 by 28 RSs; see Figure 1B, panel 5, for illustration [single trial example], and Figure 5A,B, first column, for further frequency-band-related mean FU plots) that were averaged for each individual over the respective one-second epoch trials.

The here-suggested approach tried to account for the non-linear and periodic nature of oscillatory bursts. In this approach, concrete phase and amplitude information became lost, but it was possible to quantify the average relative amount of follow-up (FU) activations per second over numerous one-second epoch trials. This information was used to explore and illustrate cross-sectional AGE-dependent changes in frequency-band-related communication dynamics (i.e., bi- and uni-directional) over the third life decade (see Figure 5A,B, third column, for the respective causal network diagrams, CNDs).

#### 2.3.4. Correlation Analyses

Absolute (see Figure 3) and relative (see Figure 4) band-pass-related regional FFT power spectrum values (for details see Section 2.3.1) were correlated (via non-parametric Spearman rank correlation) with AGE in years and several state- and trait-related covariates (see Section 2.2 for an introduction to covariates).

Average regional source (RS) moment values (mean nAm) derived from different frequency bands were correlated (via Pearson correlation) with AGE in years and several state- and trait-related covariates (see also above). Furthermore, frequency-band-related mean follow-up (FU) frequency values, as introduced in Section 2.3.3, were correlated with AGE in years. Pearson correlation coefficients, significances (second column), and causal network diagrams (CND) were illustrated in Figure 5A,B.

## 3. Results

**Figure 2:**
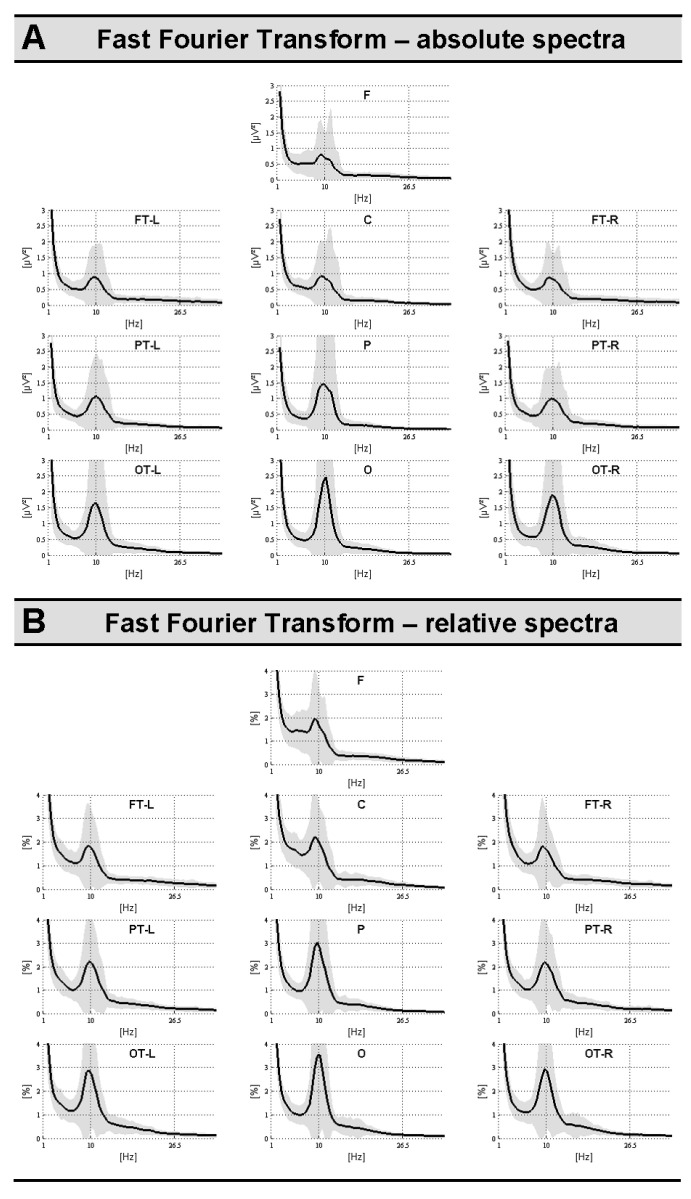
Mean regional power spectra (FFT) for absolute (panel **A**) and relative (panel **B**) spectra. Shaded areas indicate standard deviations (SDs).

Mean FFT-values and standard deviations were illustrated for absolute and relative spectra in Figure 2A,B, respectively. As expected, both absolute and relative power spectra showed a peak of around 10 Hz with regional prominence over posterior (i.e., parietal and occipital) brain areas. In the following sub-sections, correlation analyses, including non-physiological and physiological parameters, will be reported, respectively.

### 3.1. Correlation Analyses 

Neither state (i.e., PSYCH [7.6+/−1.5], PHYS [7.7+/−1.4], and STRESS [5.4+/−2.1]) nor trait (i.e., Neuroticism [1.6+/−0.6], Extraversion [2.6+/−0.6], Openness to Experience [2.8+/−0.5], Agreeableness [2.6+/−0.5], and Conscientiousness [2.6+/−0.6]) ratings revealed any significant correlative (i.e., Spearman rank correlation) relationship with AGE. Self-rated physiological (PHYS) and psychological (PSYCH) well-being was negatively correlated (PHYS: R = −0.36, *p* < 0.01; PSYCH: R = −0.41. *p* < 0.01), and stress level was positively correlated (STRESS: R = 0.28, *p* < 0.05) with AGE.

#### 3.1.1. Signal Space Analyses: Regional Absolute and Relative Power Spectra

**Figure 3:**
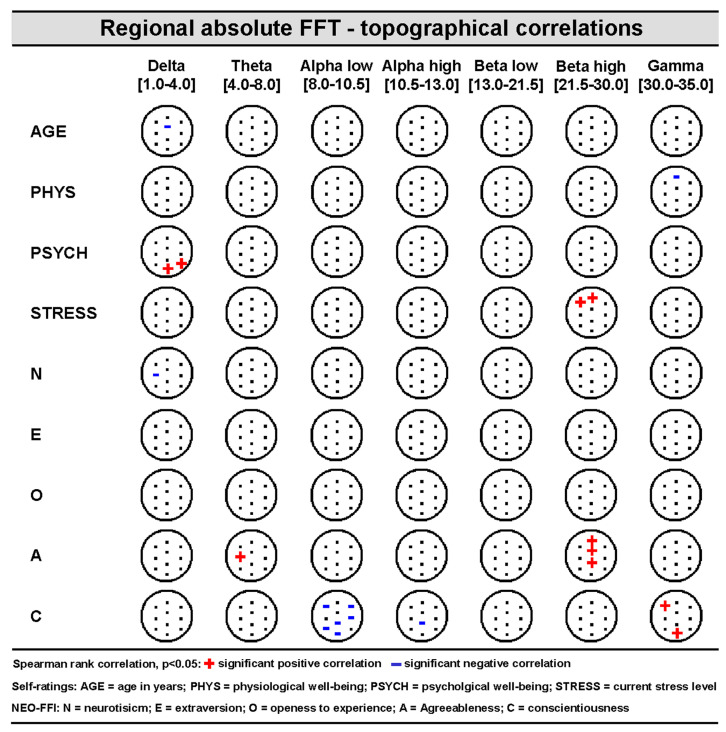
Non-parametric (Spearman) topographical rank correlation between seven absolute power spectrum frequency bands (delta, theta, alpha low, alpha high, beta low, beta high, and gamma) and nine covariates (AGE, PSYCH, PHYS, STRESS, N, E, O, V, G) (see Section 2.3.1 and legend for more details).

**Figure 4:**
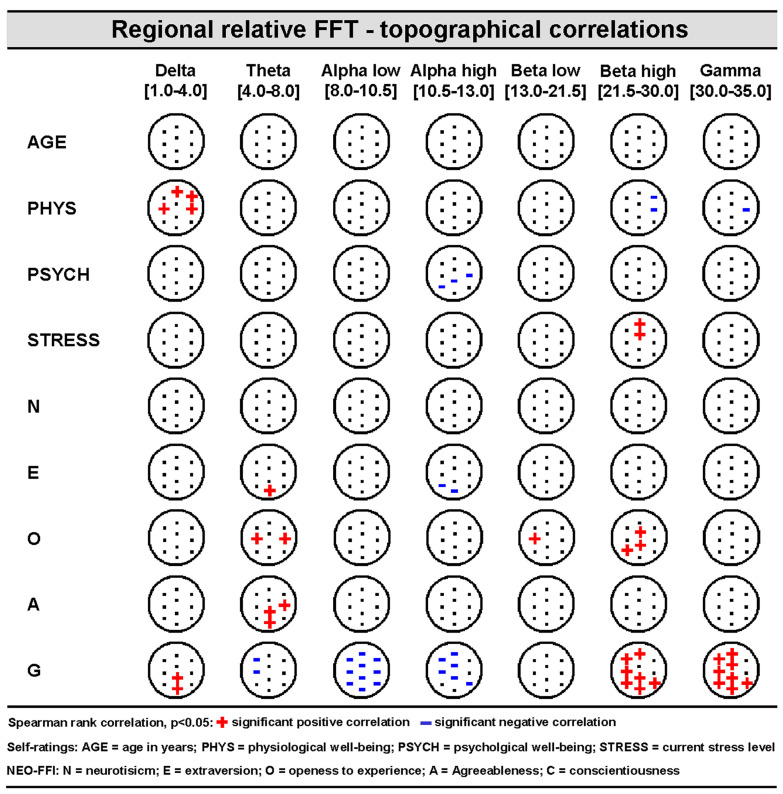
Non-parametric (Spearman) topographical rank-correlation between seven relative power spectrum frequency bands (delta, theta, alpha low, alpha high, beta low, beta high, and gamma) and nine covariates (AGE, PSYCH, PHYS, STRESS, N, E, O, V, and G) (see Section 2.3.1 and legend for more details).

Exploratory correlation analyses revealed numerous relationships between regional resting-state-related brain oscillations in different frequency bands and several state- and trait-related variables (see Figure 3 and Figure 4 for illustrations). AGE did not show any covariations with regional brain oscillatory activities in signal space. Absolute and relative regional FFT values mostly showed a different correlation profile with their respective covariates. Trait variable Conscientiousness (C) showed a comparable profile over absolute and relative regional power values, reflecting lower alpha and higher high-frequency brain activity. The self-rated stress-level appears to be related to higher beta activity over frontal brain areas.

#### 3.1.2. Source Space Analyses

Source space analyses were performed on source moment wave forms (SWFs) derived from the appliance of the source model (DMNSM) introduced in Section 2.3.2, comprising 28 regional sources (RSs, see Figure 1A, and Table 1 for details). The appliance of the DMNSM on the selected band-bass-filtered resting-state data epochs (in average 26.3 +/−5.9 one second epochs) revealed the following variance explanations: delta: 7.3+/−3.6%; theta: 3.2+/−2.2%; alpha low: 2.4+/−2.0%; alpha high: 2.7+/−3.8; beta low: 3.8+/−1.9%; beta high: 5.3+/−2.0%; gamma: 6.5+/−2.2%. One-sample *t*-test showed that all model fits ranked statistically below 10% residual variances (all *p* < 0.001). Theta and both alpha bands ranked below 5% residual variances (all *p* < 0.001).

##### Correlations between Mean Regional Source (RS) Moments and AGE

RS values did not show any significant cross-sectional correlations with AGE over the third life decade. This highly corresponded to the signal space data. There was only a trend towards significance for a negative correlation between AGE and mean delta source activity in the left medial superior frontal gyrus (lmSFG, *p* = 0.06) and left occipitotemporal junction (lOTJ, *p* = 0.07).

##### Spatiotemporal Dynamics—Follow-Up (FU) Activations and AGE-Related Causal Network Plots (CND)

**Figure 5:**
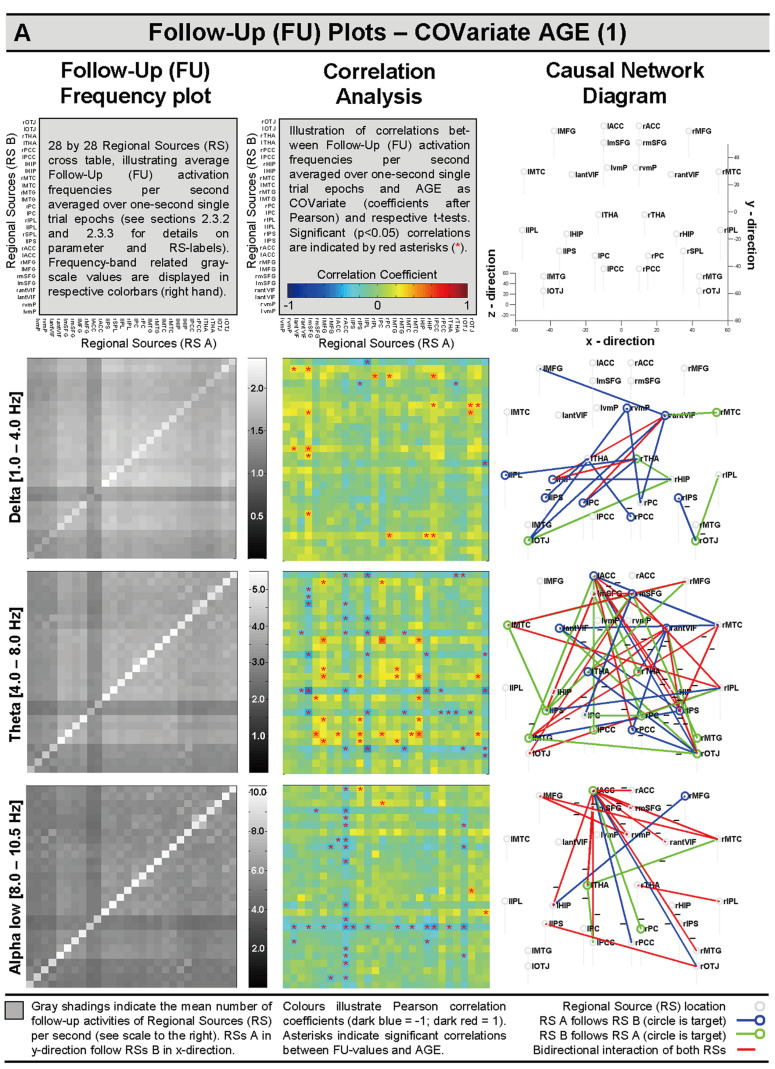

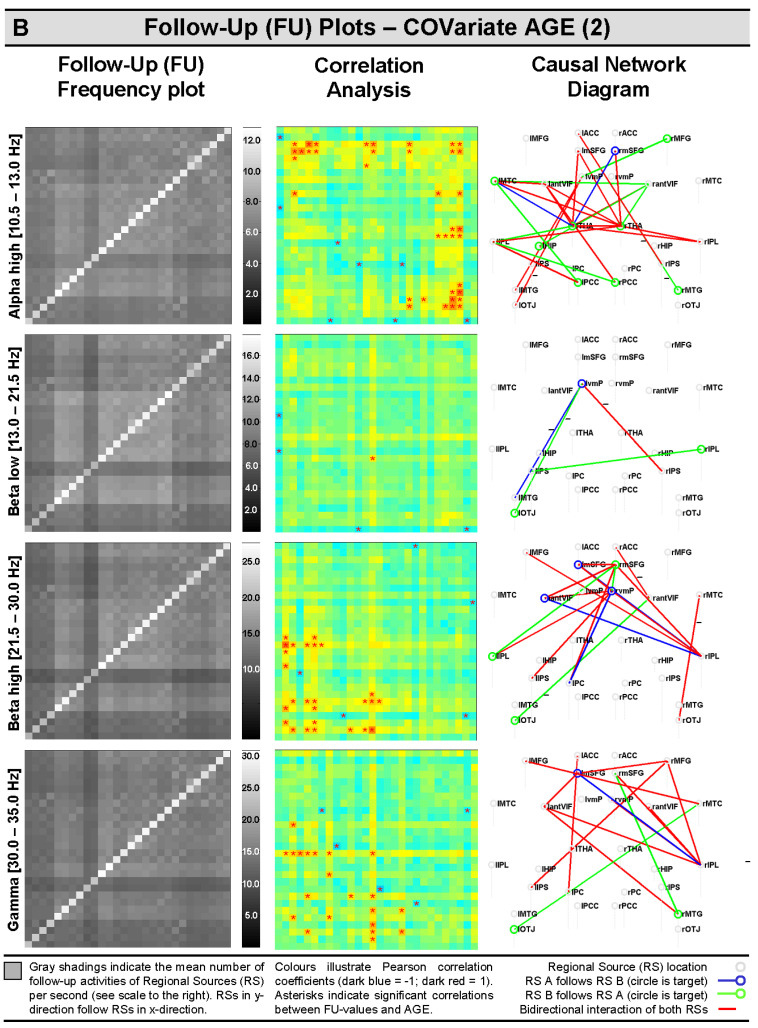
(**A**,**B**): Follow-up (FU) frequency plots (left column); heat maps for the illustration of correlations between frequency-band-related FU dynamics and AGE (middle column); and causal network diagrams (CNDs) illustrating the directionality of age-related changes between source locations of the DMNSM (right column). For more detailed information, see first panel line of (**A**), legend at the bottom, and text.

Spatiotemporal brain dynamics (as quantified by follow-up frequency calculations (see Section 2.3.3 for details) showed differential cross-sectional correlation patterns with AGE as covariate in different oscillatory frequency bands (see Figure 5A,B, second column). Follow-up frequency plots (see Figure 5A,B, first column) suggest complex patterns of regional and inter-regional source dynamics, while steady-state activations appear dominant, as indicated by relatively large values along the respective plot diagonal.

Causal network diagrams (CNDs, see Figure 5A,B, third column) indicate numerous significant (Pearson correlation, *t*-test, *p* < 0.05) positive and negative correlations, indicating both uni-directional and bi-directional neural generator dynamics cross-sectionally related to developmental changes across the third life decade. Changes in the delta oscillations suggest an age-related enhancement of uni-directional left posterior input coming from subcortical and prefrontal brain areas. The thalamus appeared to decrease its input to the left intra parietal sulcus and right posterior cingulate (see Figure 5A, third column, first CND for more details).

The CND for theta oscillations indicated a complex picture of age-related changes of positive and negative, uni- and bi-directional dynamics across the whole brain work. Numerous inter-hemispheric, uni- and bi-directional communication frequencies involving, for example, the right occipitotemporal junction, right middle temporal cortex, left middle temporal gyrus, right intra-parietal sulcus, left anterior cingulate, and left anterior ventrolateral inferior frontal areas appear to decrease their resting communication dynamics over the third life decad; whereas others, such as left medial temporal cortex, right middle temporal gyrus, right inferior parietal lobule, and left and right superior frontal gyri appear to increase their resting-state communication frequencies during the third life decade (see Figure 5A, second column, first CND for more details).

Age-related changes in communication frequencies in the lower alpha band were mainly characterized by a decrease in resting brain dynamics between the left anterior cingulate and numerous other brain areas, such as, for example, the right medial temporal cortex, left thalamus, left hippocampus, left middle frontal gyrus, and bilateral superior frontal gyri (see Figure 5A, third column, third CND for more details). Resting brain dynamics in the upper alpha band appear to predominantly increase between the left and right thalamus and several brain areas, such as, for example, the right ventromedial prefrontal areas, left and right medial superior frontal areas, right inferior parietal lobule, and left medio-temporal cortex. The left ventromedial prefrontal and left anterior cingulate areas showed a partial decrease in communication dynamics (see Figure 5B, third column, first CND for more details).

Lower beta band oscillations showed a partial age-related decrease in communication between the left ventromedial prefrontal cortex and posterior and temporal brain areas. In all, the lower beta band appears quite stable in resting-state brain dynamics during the third life decade (see Figure 5B, third column, second CND for more details). Higher beta band brain dynamics appear to increase during the third life decade predominantly within the frontal brain areas and between frontal and parietal locations mainly bi-directionally (see Figure 5B, third column, third CND for more details). Similarly, gamma band brain dynamics between 30 and 35 Hz showed an age-related increase in predominant bi-directional dynamics between frontal brain areas and in frontoparietal networks (see Figure 5B, third column, forth CND for more details).

Summarizing, the cross-sectional increases and decreases in brain dynamics over the third life decade showed a differential pattern for the seven considered oscillatory frequency bands.

## 4. Discussion

The human brain is perhaps the most complicated thing that we know of besides the universe itself. The here-presented data appear to substantiate this idea. Functional neuroimaging made it popular to examine data in a three dimensional—or cartesian-way; whereas combined with biosignal analyses (e.g., EEG and MEG), it is possible to examine spatiotemporal brain dynamics with high temporal resolution [5,13,22]. The present work relies on knowledge from functional neuroanatomy and functional neuroimaging to suggest a default network (DMN) model for the exploration of follow-up brain dynamics during the resting state in different brain oscillatory frequency bands.

First, the present signal space data confirmed the expectation that both absolute and relative power spectra should peak around 10 Hz, with dominant amplitudes over posterior sites, and that the respective resting alpha power values do not vary with age during adulthood [13,20,23,24,25,100]. Furthermore, it could be seen that personality scores did not correlate with age across the third life decade, which confirms a traditionally discussed trait-stability in personality [101,102]. The exploration of topographical brain oscillation data, considering several state and trait variables, revealed correlations between self-ratings of psychological well-being and absolute slow wave oscillations at posterior electrode sites. Ratings of stress correlated with both absolute and relative power in higher beta band over frontal areas and with age. Ratings of physical well-being correlated with relatively slow wave oscillations over the frontal and frontotemporal areas, and both self-rated psychological and physical well-being correlated negatively with age. Summarizing, signal-space data confirmed the stability of brain oscillatory activity across early adulthood [20,27,28], but it appeared to counteract MEG data that reflected a decrease in slow-wave and an increase in higher-frequency oscillations [20]. However, the sample of individuals included by Fehr et al. [20] ranged from early adulthood to elderly participants in their sixties.

The here-used regional default mode network source model (DMNSM) revealed good-to-very-good variance explanations, especially for theta and alpha frequency ranges. Mean source wave form resting-state data revealed only a statistical trend for an age-related decrease in slow wave activity, which was, however, in line with data reported by Fehr [20], who also reported a decrease in delta source activity (Minimum-Norm, L2) but also reported an increase in higher-frequency bands. It appears that age-related changes in oscillatory resting-state activation follow quite-subtle rules and cannot sufficiently be examined through just a few methodological approaches. More functional [13]—but also external—variables (e.g., individual learning history) and individual parametrization of brain physiological data [86,98,99] appears to be necessary for a sufficient explanation of age-related changes in resting-state brain activity, particularly during the third life decade.

Here, resting-state source wave form data were also examined for dynamic, oscillatory properties. The parameter that was used quantified the frequency of mean relative follow-up (FU) activations per second. FU frequency plots showed most stable activations within their respective source locations, suggesting prolonged activations within each principal region, which holds for all considered frequency bands between 1 and 35 Hz. This finding might reflect a certain relationship to microstates, discussed as a reliable measure for the occurrence of transiently stable brain activations that can, among other things, be related to temporal brain dynamics, brain developmental issues, and modalities of thinking [13,103,104,105]. In the present work, we point to the idea of counting how often certain source locations in the DMNSM follow up others and correlate this information with age (i.e., brain dynamics). The resulting partially positive and partially negative correlations showed quite different profiles, indicating age-related changes in uni-directional and bi-directional neural source sequencing for all considered frequency bands (see causal network diagrams, CNDs, Figure 5A,B). These findings suggest that there is a differential involvement of oscillatory neural networks during the development of resting-state brain activity in the third life decade. Chow and colleagues [40] also reported a non-uniform frequency depending on the involvement of different frequency oscillator dynamics as reflected in an age-related change in connectivity between the medial prefrontal and other brain areas associated with the DMN. Higher alpha connectivity decreased and both theta and beta connectivity increased with age. The here-presented CNDs confirmed growing network dynamics between the medial superior frontal and other (e.g., posterior and temporal) brain areas in higher beta and gamma bands (21.5–35 Hz) but decreased partial network dynamics in lower beta bands between the left medial prefrontal and posterior and temporal areas. Lower beta band showed rather few age-related changes in neural network dynamics, reflecting an age-related decrease in dynamics between the left ventromedial prefrontal cortex and posterior and temporal areas.

Delta oscillations showed a mainly uni-directional, age-related increase in particularly left thalamocortical network dynamics. Right anterior ventrolateral inferior location showed increasing uni-directional and bi-directional dynamics to several posterior and temporal locations. Dynamics in theta oscillation provided the most complex picture of all considered frequency bands. There were a lot of positive and negative, uni- and bi-directional dynamics across the whole DMNSM in relation to age. The predominantly involved locations were the left anterior cingulate cortex, right occipitotemporal junction, left and right intra-parietal sulci, and right anterior ventrolateral inferior frontal cortex, suggesting the involvement of executive, spatial, and object-related nodes of mental processing, potentially involved in the differential development (i.e., reorganization of related functional networks) of, for example, resting-state-related introspection and mind wandering [29,30].

Age-related changes in lower alpha dynamics appeared to be very much dominated by a decrease in follow-up communication between the left anterior cingulate cortex and numerous cortical and sub-cortical locations associated with the DMN. Thus, theta and lower alpha oscillations appear to reduce dynamic resting-state communication over the third life decade, whereas theta oscillations appear to compensate for the reduction via the establishment of new functional sub-networks. Higher alpha dynamics were dominated by an age-related increase in thalamocortical interaction, involving numerous frontal, posterior, and left temporal locations. It appears convenient to differentiate between lower and higher alpha brain dynamics.

Several limitations of the present work shall be mentioned here. Though widely accepted, the definition of the frequency band ranges used for band-pass-filtering might be revised by more fine-graded oscillatory analyses in future studies. Furthermore, longitudinal approaches provide more valid data than cross-sectional approaches. Thus, the present work shall be understood as a proof of concept that has to be approved in subsequent respective repeated examination designs. Furthermore, higher gamma band ranges above 35 Hz should also be considered.

## 5. Conclusions

Different methodological approaches often lead to apparently divergent outcomes. The present work includes traditional (i.e., topographical FFT power analyses), advanced (i.e., seeded source network analyses), and innovative approaches, exploring age-related follow-up properties in a seeded default network source model. FFT power analyses revealed the expected alpha frequency peak at about ten Hz. Topographical power analyses did not show any age-related effects, which was not completely unexpected [13]. Chow and colleagues [40] also mentioned the heterogeneity of findings on age-related resting-state examinations (e.g., [43,44,45,46]). It appears that both reliability proofs [13] and new innovative explorations are necessary for substantial and multi-perspective progress in this field. The present findings might be interpreted in such a way that quantity (i.e., power or source strength) in oscillatory resting-state processing does not reflect the most important age-related effect, but the HOW resting-state is processed [22]. According to the idea of sparse neural coding [97], quality (the reorganization of the functional HOW) might change without a change in quantity (e.g., power).

The here-introduced causal network diagrams (CNDs) shall be improved, particularly when network communications become more complicated. Video animations of respective network dynamics can help us to acquire better insights into the underlying neural processes. Furthermore, dynamic non-linear auto-correlation procedures might help not only to identify microstates [94,103,105] but also transient attractor circuits of different durations. Alongside correlative approaches, as reported in the present work, future studies should include different developmental age cohorts with different educational backgrounds, considering different experimental modalities (i.e., children, adolescents, and elderly individuals, e.g., [106,107]) and patient groups (e.g., [108]) to identify complex systemic and multi-dimensional, structural, and functional biomarkers for developmental and/or neurodegenerative diseases (e.g., [109]).

## Data Availability

As physiological data, such as multi-channel EEG recordings, have been shown to be relatable to individuals like a fingerprint and therefore bear the risk of (medical, economical) misuse, we decided to make the respective data not available online. Participants gave their written informed consent among others based on this warranty declaration. However, scientists are welcome to visit us at Bremen University and perform analyses offline in cooperation.

## References

[1] La C., Mossahebi P., Nair V.A., Bendlin B.B., Birn R., Meyerand M.E., Prabhakaran V. (2015). Age-Related Changes in Inter-Network Connectivity by Component Analysis. Front. Aging Neurosci..

[2] Thatcher R.W., Dawson G., Fischer K.W. (1994). Cyclic cortical reorganization: Origins of human cognitive development. Human Behavior and the Developing Brain.

[3] Giedd J.N., Blumenthal J., Jeffries N.O., Castellanos F.X., Liu H., Zijdenbos A., Paus T., Evans A.C., Rapoport J.L. (1999). Brain development during childhood and adolescence: A longitudinal MRI study. Nat. Neurosci..

[4] Sowell E.R., Thompson P.M., Tessner K.D., Toga A.W. (2001). Mapping continued brain growth and gray matter density reduction in dorsal frontal cortex: Inverse relationships during postadolescent brain maturation. J. Neurosci..

[5] Basar E. (2011). Brain-Body-Mind in the Nebulous Cartesian System: A Holistic Approach.

[6] Fehr T. (2009). Chancen und Grenzen von Methoden der kognitiven Neurowissenschaften—Funktionelle Magnetresonanztomographie und Biosignalanalyse im Kontext der Entwicklungsneurophysiologie. Z. Gestalt..

[7] Fehr T., Martin C.R., Preedy V.R., Patel V.B. (2023). The Neural Architecture of Violence-Related Socialization—Evidence from Functional Neuroimaging. Handbook of Anger, Aggression, and Violence.

[8] Lorente de No R. (1947). A Study of Nerve Physiology: Studies from the Rockefeller Institute of Medical Research.

[9] Basar E. (2006). The theory of the whole-brain-work. Int. J. Psychophysiol..

[10] Weiss A., Faber D.S. (2010). Field effects in the CNS play functional roles. Front. Neural Circuits.

[11] Hindriks R., Woolrich M., Luckhoo H., Joensson M., Mohseni H., Kringelbach M.L., Deco G. (2015). Role of white-matter pathways in coordinating alpha oscillations in resting visual cortex. Neuroimage.

[12] Babiloni C., Del Percio C., Boccardi M., Lizio R., Lopez S., Carducci F., Marzano N., Soricelli A., Ferri R., Triggiani A.I. (2015). Occipital sources of resting-state alpha rhythms are related to local gray matter density in subjects with amnesic mild cognitive impairment and Alzheimer’s disease. Neurobiol. Aging.

[13] Popov T., Tröndle M., Baranczuk-Turska Z., Pfeiffer C., Haufe S., Langer N. (2023). Test–retest reliability of resting-state EEG in young and older adults. Psychophysiology.

[14] Scherg M., Berg P., Nakasato N., Beniczky S. (2019). Taking the EEG back into the brain: The power of multiple discrete sources. Front. Neurol..

[15] Scherg M. (1992). Functional imaging and localization of electromagnetic brain activity. Brain Topogr..

[16] Scherg M., Berg P. (1996). New concepts of brain source imaging and localization. Electroencephalogr. Clin. Neurophysiol. Suppl..

[17] Scherg M., Berg P. (1991). Use of prior knowledge in brain electromagnetic source analysis. Brain Topogr..

[18] Scherg M. (2004). BESA Source Analysis Combining EEG and fMRI. E-Book. www.besa.de.

[19] Somsen R.J., van’t Klooster B.J., van der Molen M.W., van Leeuwen H.M., Licht R. (1997). Growth spurts in brain maturation during middle childhood as indexed by EEG power spectra. Biol. Psychol..

[20] Fehr T., Bott C., Haeberle A., Rockstroh B., Nowak H., Haueisen J., Gießler F., Huonker R. (2002). MEG power spectrum and age: Differences between adolescents and adults. BIOMAG 2002—13th International Conference on Biomagnetism, Jena, Germany, 10–14 August 2002.

[21] Finley A.J., Angus D.J., van Reekum C.M., Davidson R.J., Schaefer S.M. (2022). Periodic and aperiodic contributions to theta-beta ratios across adulthood. Psychophysiology.

[22] Fehr T. (2013). A hybrid model for the neural representation of complex mental processing in the human brain. Cogn. Neurodyn..

[23] Basar E. (2012). A review of alpha activity in integrative brain function: Fundamental physiology, sensory coding, cognition and pathology. Int. J. Psychophysiol..

[24] Adrian E.D., Matthews B.H.C. (1934). The Berger rhythm: Potential changes from the occipital lobes in man. Brain.

[25] Compston A. (2010). The Berger rhythm: Potential changes from the occipital lobes in man. Brain.

[26] Grandy T.H., Werkle-Bergner M., Chicherio C., Schmiedek F., Lovden M., Lindenberger U. (2013). Peak individual alpha frequency qualifies as a stable neurophysiological trait marker in healthy younger and older adults. Psychophysiology.

[27] Cragg L., Kovacevic N., McIntosh A.R., Poulsen C., Martinu K., Leonard G., Paus T. (2011). Maturation of EEG power spectra in early adolescence: A longitudinal study. Dev. Sci..

[28] Miskovic V., Ma X., Chou C.A., Fan M., Owens M., Sayama H., Gibb B.E. (2015). Developmental changes in spontaneous electrocortical activity and network organization from early to late childhood. Neuroimage.

[29] Mason M.F., Norton M.I., Van Horn J.D., Wegner D.M., Grafton S.T., Macrae C.N. (2007). Wandering minds: The default network and stimulus-independent thought. Science.

[30] Andrews-Hanna J.R. (2012). The Brain’s default network and its adaptive role in internal mentation. Neuroscientist.

[31] Buckner R.L., Andrews-Hanna J.R., Schacter D.L. (2008). The brain’s default network: Anatomy, function, and relevance to disease. Ann. N. Y. Acad. Sci..

[32] Wen T., Mitchell D.J., Duncan J. (2020). The functional convergence and heterogeneity of social, episodic, and self-referential thought in the default mode network. Cereb. Cortex.

[33] Anticevic A., Cole M.W., Murray J.D., Corlett P.R., Wang X.-J., Krystal J.H. (2012). The role of default network deactivation in cognition and disease. Trends Cogn. Sci..

[34] Beckmann C.F., Arigita E.J.S., Barkhof F., Scheltens P., Stam C.J., Smith S.M., Rombouts S.A.R.B. (2008). Reduced resting-state brain activity in the ‘‘default network” in normal aging. Cereb. Cortex.

[35] Hafkemeijer A., van der Grond J., Rombouts S.A.R.B. (2012). Imaging the default mode network in aging and dementia. Biochim. Biophys. Acta Mol. Basis Dis..

[36] Campbell K., Grigg O., Saverino C., Churchill N., Grady C. (2013). Age differences in the intrinsic functional connectivity of default network subsystems. Front. Aging Neurosci..

[37] Andrews-Hanna J.R., Snyder A.Z., Vincent J.L., Lustig C., Head D., Raichle M.E., Buckner R.L. (2007). Disruption of large-scale brain systems in advanced aging. Neuron.

[38] Persson J., Lustig C., Nelson J.K., Reuter-Lorenz P.A. (2007). Age differences in deactivation: A link to cognitive control?. J. Cogn. Neurosci..

[39] Grady C.L., Protzner A.B., Kovacevic N., Strother S.C., Afshin-Pour B., Wojtowicz M., Anderson J.A.E., Churchill N., McIntosh A.R. (2010). A Multivariate analysis of age-related differences in default mode and task-positive networks across multiple cognitive domains. Cereb. Cortex.

[40] Chow R., Rabi R., Paracha S., Hasher L., Anderson N.D., Alain C. (2022). Default Mode Network and Neural Phase Synchronization in Healthy Aging: A Resting State EEG Study. Neuroscience.

[41] Cabral J., Kringelbach M.L., Deco G. (2014). Exploring the network dynamics underlying brain activity during rest. Prog. Neurobiol..

[42] Ghuman A.S., McDaniel J.R., Martin A. (2011). A wavelet-based method for measuring the oscillatory dynamics of resting-state functional connectivity in MEG. Neuroimage.

[43] Vysata O., Kukal J., Prochazka A., Pazdera L., Simko J., Valis M. (2014). Age-related changes in EEG coherence. Neurol. Neurochir. Pol..

[44] Scally B., Burke M.R., Bunce D., Delvenne J.-F. (2018). Resting-state EEG power and connectivity are associated with alpha peak frequency slowing in healthy aging. Neurobiol. Aging.

[45] Smit D.J.A., Boersma M., Schnack H.G., Micheloyannis S., Boomsma D.I., Hulshoff Pol H.E., Stam C.J., de Geus E.J.C., Valdes-Sosa P.A. (2012). The brain matures with stronger functional connectivity and decreased randomness of its network. PLoS ONE.

[46] Moezzi B., Pratti L.M., Hordacre B., Graetz L., Berryman C., Lavrencic L.M., Ridding M.C., Keage H.A.D., McDonnell M.D., Goldsworthy M.R. (2019). Characterization of young and old adult brains: An EEG functional connectivity analysis. Neuroscience.

[47] Kikuchi M., Wada Y., Koshino Y., Nanbu Y., Hashimoto T. (2000). Effect of Normal Aging upon Interhemispheric EEG Coherence: Analysis during Rest and Photic Stimulation. Clin. Electroencephalogr..

[48] Vecchio F., Miraglia F., Bramanti P., Rossini P.M. (2014). Human brain networks in physiological aging: A graph theoretical analysis of cortical connectivity from EEG data. J. Alzheimer’s Dis..

[49] Von Bonin G.V. (1950). Essay on the Cerebral Cortex.

[50] Fuster J.M. (2006). The cognit: A network model of cortical representation. Int. J. Psychophysiol..

[51] Fuster J.M. (2009). Cortex and Memory: Emergence of a New Paradigm. J. Cogn. Neurosci..

[52] van Oort E.S., Van Walsum A.V.C., Norris D.G. (2014). An investigation into the functional and structural connectivity of the Default Mode Network. Neuroimage.

[53] Van Den Heuvel M.P., Sporns O. (2011). Rich-club organization of the human connectome. J. Neurosci..

[54] Achard S., Salvador R., Whitcher B., Suckling J., Bullmore E. (2006). A resilient, low-frequency, small-world human brain functional network with highly connected association cortical hubs. J. Neurosci..

[55] Hellyer P.J., Shanahan M., Scott G., Wise R.J., Sharp D.J., Leech R. (2014). The control of global brain dynamics: Opposing actions of frontoparietal control and default mode networks on attention. J. Neurosci..

[56] Avelar-Pereira B., Bäckman L., Wåhlin A., Nyberg L., Salami A. (2017). Age-related differences in dynamic interactions among default mode, frontoparietal control, and dorsal attention networks during resting-state and interference resolution. Front. Aging Neurosci..

[57] Menon V. (2023). 20 years of the default mode network: A review and synthesis. Neuron.

[58] Di Plinio S., Ferri F., Marzet L., Romani G.L., Northoff G., Pizzella V. (2018). Functional connections between activated and deactivated brain regions mediate emotional interference during externally directed cognition. Hum. Brain Mapp..

[59] Uddin L.Q., Kelly A.M., Biswal B.B., Castellanos F.X., Milham M.P. (2009). Functional connectivity of default mode network components: Correlation, anticorrelation, and causality. Hum. Brain Mapp..

[60] Konu D., Turnbull A., Karapanagiotidis T., Wang H.-T., Brown L.R., Jefferies E., Smallwood J. (2020). A role for the ventromedial prefrontal cortex in self-generated episodic social cognition. Neuroimage.

[61] Alves P.N., Foulon C., Karolis V., Bzdok D., Margulies D.S., Volle E., de Schotten M.T. (2019). An improved neuroanatomical model of the default-mode network reconciles previous neuroimaging and neuropathological findings. Commun. Biol.

[62] Binder J.R., Frost J.A., Hammeke T.A., Bellgowan P., Rao S.M., Cox R.W. (1999). Conceptual processing during the conscious resting state: A functional MRI study. J. Cogn. Neurosci..

[63] Damoiseaux J.S., Rombouts S.A.R.B., Barkhof F., Scheltens P., Stam C.J., Smith S.M., Beckmann C.F. (2006). Consistent resting-state networks across healthy subjects. Proc. Natl. Acad. Sci. USA.

[64] Greicius M.D., Krasnow B., Reiss A.L., Menon V. (2003). Functional connectivity in the resting brain: A network analysis of the default mode hypothesis. Proc. Natl. Acad. Sci. USA.

[65] Koch W., Teipel S., Mueller S., Buerger K., Bokde A., Hampel H., Coates U., Reiser M., Meindl T. (2010). Effects of aging on default mode network activity in resting state fMRI: Does the method of analysis matter?. Neuroimage.

[66] Schilbach L., Eickhoff S.B., Rotarska-Jagiela A., Fink G.R., Vogeley K. (2008). Minds at rest? Social cognition as the default mode of cognizing and its putative relationship to the “default system” of the brain. Conscious. Cogn..

[67] Bluhm R.L., Osuch E.A., Lanius R.A., Boksman K., Neufeld R.W., Théberge J., Williamson P. (2008). Default mode network connectivity: Effects of age, sex, and analytic approach. Neuroreport.

[68] Das A., de Los Angeles C., Menon V. (2022). Electrophysiological foundations of the human default-mode network revealed by intracranial-EEG recordings during resting state and cognition. Neuroimage.

[69] Dixon M.L., Moodie C.A., Goldin P.R., Farb N., Heimberg R.G., Zhang J., Gross J.J. (2021). Frontoparietal and default mode network contributions to self-referential processing in social anxiety disorder. Cogn. Affect. Behav. Neurosci..

[70] Laird A.R., Eickhoff S.B., Li K., Robin D.A., Glahn D.C., Fox P.T. (2009). Investigating the functional heterogeneity of the default mode network using coordinate-based meta-analytic modeling. J. Neurosci..

[71] DeSerisy M., Ramphal B., Pagliaccio D., Raffanello E., Tau G., Marsh R., Posner J., Margolis A.E. (2021). Frontoparietal and default mode network connectivity varies with age and intelligence. Dev. Cogn. Neurosci..

[72] Davey C.G., Pujol J., Harrison B.J. (2016). Mapping the self in the brain’s default mode network. Neuroimage.

[73] Binkofski F.C., Klann J., Caspers S. (2016). Chapter 4—On the Neuroanatomy and Functional Role of the Inferior Parietal Lobule and Intraparietal Sulcus. Neurobiology of Language.

[74] Mars R.B., Jbabdi S., Sallet J., O’Reilly J.X., Croxson P.L., Olivier E., Noonan M.P., Bergmann C., Mitchell A.S., Baxter M.G. (2011). Diffusionweighted imaging tractography-based parcellation of the human parietal cortex and comparison with human and macaque resting-state functional connectivity. J. Neurosci..

[75] Thomas Yeo B.T., Krienen F.M., Sepulcre J., Sabuncu M.R., Lashkari D., Hollinshead M., Roffman J.L., Smoller J.W., Zöllei L., Polimeni J.R. (2011). The organization of the human cerebral cortex estimated by intrinsic functional connectivity. J. Neurophysiol..

[76] Wu S.-C.J., Jenkins L.M., Apple A.C., Petersen J., Xiao F., Wang L., Yang F.-P.G. (2020). Longitudinal fMRI task reveals neural plasticity in default mode network with disrupted executive-default coupling and selective attention after traumatic brain injury. Brain Imaging Behav..

[77] Smallwood J., Bernhardt B.C., Leech R., Bzdok D., Jefferies E., Margulies D.S. (2021). The default mode network in cognition: A topographical perspective. Nat. Rev. Neurosci..

[78] Briggs R.G., Tanglay O., Dadario N.B., Young I.M., Fonseka R.D., Hormovas J., Dhanaraj V., Lin Y.-H., Kim S.J., Bouvette A. (2021). The unique fiber anatomy of middle temporal gyrus default mode connectivity. Oper. Neurosurg..

[79] Xu J., Wang J., Fan L., Li H., Zhang W., Hu Q., Jiang T. (2015). Tractography-based parcellation of the human middle temporal gyrus. Sci. Rep..

[80] Cunningham S.I., Tomasi D., Volkow N.D. (2017). Structural and functional connectivity of the precuneus and thalamus to the default mode network. Hum. Brain Mapp..

[81] Lee T.W., Xue S.W. (2018). Functional connectivity maps based on hippocampal and thalamic dynamics may account for the default-mode network. Eur. J. Neurosci..

[82] Sontheimer A., Pontier B., Claise B., Chassain C., Coste J., Lemaire J.-J. (2021). Disrupted pallido-thalamo-cortical functional connectivity in chronic disorders of consciousness. Brain Sci..

[83] Yuan R., Di X., Taylor P.A., Gohel S., Tsai Y.-H., Biswal B.B. (2016). Functional topography of the thalamocortical system in human. Brain Struct. Funct..

[84] Trepel M. (2021). Neuroanatomie: Struktur und Funktion.

[85] Fodor J. (1983). The Modularity of Mind.

[86] Fehr T., Milz P. (2019). The individuality index—A measure to quantify the degree of inter-individual variability in intra-cerebral brain electric and metabolic activity. Cogn. Neurodyn..

[87] Oldfield R.C. (1997). The assessment and analysis of handedness: The Edinburgh inventory. Neuropsychologia.

[88] Costa P.T., McCrae R.R. (1992). Revised NEO Personality Inventory and NEO Five-Factor Inventory.

[89] Borkenau P., Ostendorf F. (1993). NEO-Fünf-Faktoren Inventar (NEO-FFI): Nach Costa und McCrae.

[90] Fehr T., Achtziger A. (2021). Contextual modulation of binary decisions in dyadic social interactions. Front. Behav. Neurosci..

[91] Ille N., Berg P., Scherg M. (2002). Artifact correction of the ongoing EEG using spatial filters based on artifact and brain signal topographies. J. Clin. Neurophysiol..

[92] Talairach J., Tournoux P. (1988). Co-Planar Stereotaxic Atlas of the Human Brain.

[93] Hopfinger J., Khoe W., Song A., Handy T.C. (2005). Combining Electrophysiology with structural and functional Neuroimaging: ERP’s, PET, MRI, fMRI. Event-Related Potential: A Methods Handbook.

[94] Michel C.M., Koenig T. (2018). EEG microstates as a tool for studying the temporal dynamics of whole-brain neuronal networks: A review. NeuroImage.

[95] Bledowski C., Prvulovic D., Hoechstetter K., Scherg M., Wibral M., Goebel R., Linden D.E. (2004). Localizing P300 generators in visual target and distractor processing: A combined event-related potential and functional magnetic resonance imaging study. J. Neurosci..

[96] Buckner R.L., Di Nicola L.M. (2019). The brain’s default network: Updated anatomy, physiology and evolving insights. Nat. Rev. Neurosci..

[97] Palm G. (2013). Neural associative memories and sparse coding. Neural Netw..

[98] Fehr T., Achtziger A., Hinrichs H., Herrmann M., Reinvang I., Greenlee M.W., Herrmann M. (2003). Interindividual differences in oscillatory brain activity in higher cognitive functions—Methodological approaches in analyzing continuous MEG data. The Cognitive Neuroscience of Individual Differences.

[99] Achtziger A., Fehr T., Oettingen G., Gollwitzer P., Rockstroh B. (2008). Strategies of Intention Formation are Reflected in Continuous MEG Activity. Soc. Neurosci..

[100] Fehr T. (2002). Lokalisation Langsamer Hirnaktivität bei Schizophrenen Patienten Mittels Magnetenzephalografischer Untersuchungen und Exploration von Zusammenhängen Zwischen Langsamwelliger Hirnaktivität und Symptomatik.

[101] Hampson S.E., Goldberg L.R., Carducci B.J., Nave C.S. (2021). Personality Stability and Change over Time. The Wiley Encyclopedia of Personality and Individual Differences: Models and Theories.

[102] Zhi S., Zhao W., Wang R., Li Y., Wang X., Liu S., Li J., Xu Y. (2023). Stability of specific personality network features corresponding to openness trait across different adult age periods: A machine learning analysis. Biochem. Biophys. Res. Commun..

[103] Koenig T., Prichep L., Lehmann D., Sosa P.V., Braeker E., Kleinlogel H., Isenhart R., John E.R. (2002). Millisecond by millisecond, year by year: Normative EEG microstates and developmental stages. NeuroImage.

[104] Michel C.M., Murray M.M., Lantz G., Gonzalez S., Spinelli L., de Peralta R.G. (2004). EEG source imaging. Clin. Neurophysiol..

[105] Milz P., Faber P.L., Lehmann D., Koenig T., Kochi K., Pascual-Marqui R.D. (2016). The functional significance of EEG microstates--associations with modalities of thinking. NeuroImage.

[106] Stoffel T., Vaque-Alcazar L., Bartres-Faz D., Pero-Cebollero M., Canete-Masse C., Guardia-Olmos J. (2024). Reduced default mode network effective connectivity in healthy aging is modulated by years of education. NeuroImage.

[107] Giannopoulos A.E., Zioga I., Papageorgiou P., Pervanidou P., Makris G., Chrousos G.P., Stachtea X., Capsalis C., Papageorgiou C. (2022). Evaluating the Modulation of the Acoustic Startle Reflex in Children and Adolescents via Vertical EOG and EEG: Sex, Age, and Behavioral Effects. Front. Psychol..

[108] Giustiniani A., Danesin L., Bozzetto B., Macina A., Benavides-Varela S., Burgio F. (2023). Functional changes in brain oscillations in dementia: A review. Rev. Neurosci..

[109] Moreno-De-Luca A., Myers S.M., Challman T.D., Moreno-De-Luca D., Evans D.W., Ledbetter D.H. (2013). Developmental brain dysfunction: Revival and expansion of old concepts based on new genetic evidence. Lancet Neurol..

